# Clinical Audit to Assess Upper Airway Ultrasound Skills in Anaesthesia Trainees

**DOI:** 10.7759/cureus.7272

**Published:** 2020-03-14

**Authors:** Atul A Ambekar, Jan Stevens

**Affiliations:** 1 Anaesthesiology, Ipswich Hospital, East Suffolk and North Essex NHS Foundation Trust, Ipswich, GBR

**Keywords:** upper airway ultrasound, training, clinical audit

## Abstract

This audit looks at upper airway ultrasound skills and basic knowledge of anaesthesia trainees. The implementation took place in a District General Hospital in the United Kingdom where upper airway ultrasound is not a part of formal training. Seventeen anaesthetic trainees were given hands-on experience of upper airway ultrasound and were asked to fill in a questionnaire. The result showed a rapid learning curve for assessing cricothyroid membrane localisation, but difficulty in oesophageal identification. A potential plan to improve this skill was proposed and if implemented, will help all trainee anaesthetists in the present and future to develop this skill.

## Introduction

Ultrasound is an established tool for peripheral nerve blockade, difficult peripheral venous cannulation, central venous cannulation, difficult arterial cannulation, central neuraxial blockade in obese and pathological spines. Upper airway ultrasound is a skill that is yet to become universal amongst the anaesthesia community. Various studies have been performed and have shown that upper airway ultrasound is beneficial in predicting difficult laryngoscopy, predicting the size of endotracheal tube, identifying correct endotracheal and laryngeal mask airway placement, identifying the cricothyroid membrane, prediction of post-extubation stridor and evaluation of the epiglottis [1-3].

## Materials and methods

This project was exempted from Institutional Review Board approval as it was a local departmental clinical audit project involving non-invasive methodology with primary focus on developing training tools for the trainees. Seventeen anaesthesiology trainees, which included 12 core trainees, three speciality trainees, and two speciality registrars, were given hands-on experience of upper airway ultrasound with a Sonosite Edge Ultrasound machine. The trainees rotated amongst themselves as subjects for the ultrasound examination. Each trainee got an opportunity to scan twice. We explained and guided each trainee during the first scan, and then the trainees did the second scan independently. The trainees identified various structures like base of tongue, epiglottis, hyoid bone, vocal cords, skin to air-mucosal interface distance, thyroid cartilage, cricoid cartilage, cricothyroid membrane, oesophagus and trachea. The total duration of the first scan was five minutes and second scan was three minutes on an average. Then the trainees were provided with a questionnaire to fill in. The questionnaire was prepared and validated by a team of one consultant anaesthetist, one speciality trainee, and one speciality registrar. We used a eleven-point Likert scale to measure the responses as it has more sensitivity as compared to a three, five or seven-point Likert scale [4].

The questionnaire was as follows:

1. How easy was it to perform upper airway ultrasound in general?

2. How confident are you that you can identify the cricothyroid membrane?

3. How confident are you in identifying the vocal cords on ultrasound and measuring the distance from skin to air-mucosal interface to predict difficult intubation?

4. How confident are you in identifying the trachea on ultrasound?

5. Could you identify oesophageal intubation with ultrasound at the level of the thyroid gland?

The answers were based on a score of zero - ten, zero being not confident and ten being highly confident. Grossly scores zero to four would represent low confidence, scores five to seven would represent moderate confidence, and scores eight to ten would represent high confidence.

## Results

Most of the trainees expressed reasonable ease to perform upper airway ultrasound in general, the overall confidence score range being five to ten, with a maximum of six responses for a confidence score of eight. The trainees could quite confidently identify cricothyroid membrane and trachea with scores ranging from six to nine and six to ten respectively. Identification of vocal cords and skin to air-mucosal interface had a more varied confidence score ranging from four to ten. Identification of oesophagus was by far the most difficult for all trainees, with a confidence score ranging from zero to ten, maximum of six responses for a score of four and two outliers - one for a score of zero and one for a score of ten (Table 1).

**Table 1:** Results of upper airway ultrasound questionnaire

Parameter	Confidence score range expressed by trainees	Maximum responses for a score of
Ease of performance	5 – 10	8 (6 responses)
Identification of cricothyroid membrane	6 – 9	6 (8 responses)
Identification of vocal cords and skin to air-mucosal interface distance	4 – 10	7 & 8 (4 responses each)
Identification of trachea	6 – 10	9 (6 responses)
Identification of oesophagus	0 – 10	6 (4 responses)

Irrespective of the level of anaesthesia experience of trainees, the trend was similar. In general, most of the trainees found identification of cricothyroid membrane and trachea relatively easy, and identification of oesophagus the most difficult.

## Discussion

Upper airway ultrasound is a valuable point-of-care tool to assess airway anatomy. Various structures such as base of the tongue, epiglottis, hyoid bone, vocal cords, thyroid cartilage, cricoid cartilage, cricothyroid membrane, and oesophagus can be seen on the sono-anatomy. Developing this skill will help the anaesthetist to assess the airway for difficult intubation, confirming the placement of endotracheal tube into the trachea, predicting the appropriate size of laryngeal mask airway and endotracheal tube, identifying oesophageal intubation, and identifying the cricothyroid membrane for front of neck access [1].

Cricothyroid membrane has special importance in anaesthesia especially in obese, pregnant and difficult airway patients. Identification of the cricothyroid membrane in such patients with an ultrasound prior to induction of anaesthesia can prove beneficial if there arises a situation where a front of neck access needs to be secured. Palpation of cricothyroid membrane is limited by factors like thick pad of subcutaneous tissue, obesity and in females due to the relatively less palpable thyroid prominence. Palpation is also limited by distorted anatomy due to previous radiotherapy to neck and tracheal deviation [5]. A computed tomography (CT)-guided study demonstrated that cricothyroid membrane is not necessarily a superficial structure and also the smallest dimensions of the membrane may necessitate the use of smaller than recommended trocar and endotracheal tubes [6]. A study by Kristensen et al. demonstrated 37% success rate with conventional digital palpation method with a mean time of 18 seconds, and 83% success rate with a structured stepwise ultrasonography method with a mean time of 48 seconds [7].

This project aimed to explore the understanding of upper airway ultrasound in the trainees and provide them an opportunity to perform one, which could stimulate their interest and help them make upper airway ultrasound an important tool in their armamentarium. The results indicate that the overall confidence of trainees is average when it comes to upper airway ultrasound in general but good when identifying the cricothyroid membrane. Also the level of anaesthesia experience of the trainee had little effect on the confidence score, highlighting the fact that upper airway ultrasound is not a routinely practiced activity in this hospital.

We propose the following for the trainees, to develop and improve their upper airway ultrasound skills:

1. Use of upper airway ultrasound to predict difficult intubation, initially in normal airway patients, and then in obese and difficult airway patients

2. Use of upper airway ultrasound to identify cricothyroid membrane before embarking on intubation, especially in obese and difficult airway patients

3. Use of upper airway ultrasound to identify correct placement of endotracheal tube i.e. to rule out oesophageal and endobronchial intubation

We recommend that a pre-anaesthetic assessment of potentially difficult airway patients be regularly done, wherein a timely upper airway ultrasound can be scheduled. This will provide the trainees an opportunity to perform the same under consultant supervision. Also the trainees can then follow-up the patient and do an upper airway ultrasound in the theatre post-induction to verify the placement of the airway device. This timely scheduling of the ultrasound will avoid loss of theatre time before induction and the post-induction ultrasound can usually be done while the surgical preparations commence.

## Conclusions

This project highlights the importance of identifying vital airway structures in potentially difficult airway patients using ultrasound. It also highlights the lack of a formal upper airway ultrasound training structure for trainees and the consequent average confidence of trainees with respect to the same. It paves way for introduction of formal training of this vital skill and also stimulates the trainees to take up similar clinical audits and research projects to increase their breadth of knowledge and skill about upper airway ultrasound.

## References

[1] Osman A, Sum KM (2016). Role of upper airway ultrasound in airway management. J Intensive Care.

[2] Lakhal K, Delplace X, Cottier JP (2007). The feasibility of ultrasound to assess subglottic diameter. Anesth Analg.

[3] Adhikari S, Zeger W, Schmier C (2011). Pilot study to determine the utility of point-of-care ultrasound in the assessment of difﬁcult laryngoscopy. Acad Emerg Med.

[4] Leung S (2011). A comparison of psychometric properties and normality in 4-, 5-, 6-, and 11-point Likert scales. J Soc Serv Res.

[5] Campbell M, Shanahan H, Ash S, Royds J, Husarova V, McCaul C (2014). The accuracy of locating the cricothyroid membrane by palpation - an inter-gender study. BMC Anesthesiol.

[6] Long N, Ng S, Donnelly G, Owens M, McNicholas M, McCarthy K, McCaul C (2014). Anatomical characterisation of the cricothyroid membrane in females of childbearing age using computed tomography. Int J Obstet Anesth.

[7] Kristensen MS, Teoh WH, Rudolph SS (2015). Structured approach to ultrasound-guided identification of the cricothyroid membrane: a randomized comparison with the palpation method in the morbidly obese. Br J Anaesth.

